# Dislocated Extensor Mechanism in a Child With Bilateral Absent Patella Without Other Congenital Abnormalities

**DOI:** 10.5435/JAAOSGlobal-D-23-00117

**Published:** 2023-09-15

**Authors:** Austin Yu, Marina Russo, Deepika Kothakapa, Patrick Huang, Abigail Mantica

**Affiliations:** From the Division of Orthopaedic Surgery, Albany Medical Center, Albany, NY.

## Abstract

**Case:**

A 10-year-old boy presented with bilateral absent patella and dislocation of the extensor mechanism in the left lower extremity. He underwent a lateral release and medial plication of the extensor mechanism with a Roux-Goldthwait procedure, followed by casting and bracing treatment. The patient fully recovered with a return to sports and improved active range of motion.

**Conclusion:**

Bilateral absent patella without other congenital anomalies is an exceedingly rare condition and can be accompanied by a dislocation of the extensor mechanism. Treatment should address functional limitations, including extensor mechanism dislocation, when present.

Patellar aplasia or hypoplasia is a rare congenital condition and usually associated with multiple syndromes, including nail-patella syndrome, small patella syndrome, Meier-Gorlin syndrome, RAPADILINO syndrome, trisomy 8, and others.^1–3^ Absence of bilateral patellas without other congenital anomalies is extremely rare with only a few previous reports.^2–4^ Absent patella or hypoplastic patella may be accompanied by dislocation of the patella or the extensor mechanism, leading to weak or absent knee extension.^4–6^ To our knowledge, this is the first report of an extensor mechanism dislocation in the context of bilateral absent patella without other congenital anomalies.

IRB exemption was attained, and consent was determined not necessary for this case study.

## Case Report

A 10-year-old boy was referred to the pediatric orthopaedic clinic for evaluation of an acute gait abnormality. Approximately 5 months before evaluation, the patient suddenly started limping and dragging his left lower extremity behind him. He denied any trauma and was participating in sports before this incident. He was also unable to straighten his knee from a flexed position and was no longer able to run. He was initially seen by his primary care provider and was prescribed a course of physical therapy, which did not improve his symptoms. The patient was otherwise healthy, and there was no other family history of any bone dysplasias or any other skeletal abnormalities. The patient met all his developmental milestones and had not undergone any genetic testing.

On examination, the patient was unable to initiate extension of the left knee when it was in a flexed position. He could keep his left knee extended after passive extension of the knee. He had full active range of motion of the right knee and could actively extend his right knee against resistance. No instability was noted in either knee on Lachman, posterior drawer, and varus and valgus stress tests. No palpable patella was noted on either side. Both legs were negative for knee effusion and were not tender to palpation.

**Figure 1:**
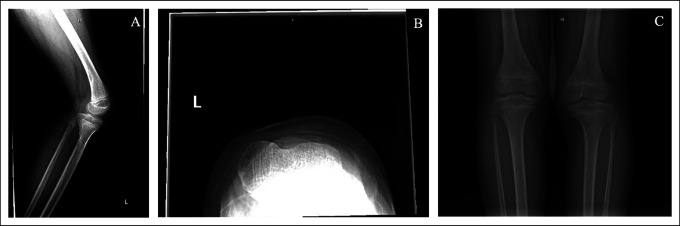
Radiograph images demonstrating bilateral absent patella. **A,** Left knee lateral view, (**B**) left knee sunrise view, and (**C**) bilateral weight-bearing AP view.

**Figure 2:**
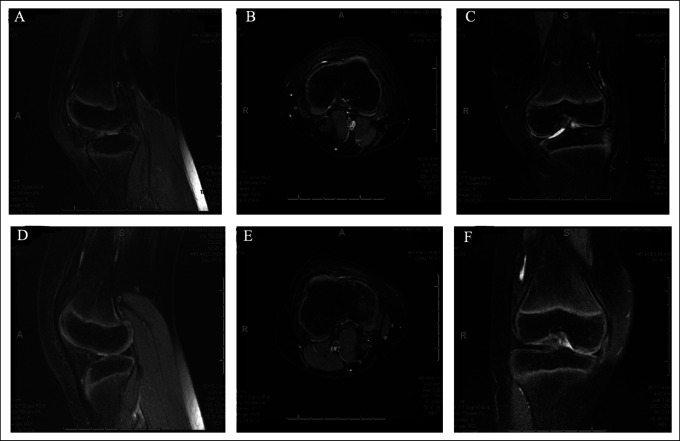
T2-weighted MRI scans of the right and left knee demonstrating bilateral absent patella. The rest of the ligamentous structures were intact. **A,** Left knee sagittal section, (**B**) left knee axial section, (**C**) left knee coronal section, (**D**) right knee sagittal section, (**E**) right knee axial section, and (**F**) right knee coronal section. MRI = magnetic resonance imaging.

Radiographs confirmed the absence of patellas in both knees (Figure 1). Both knees were evaluated with magnetic resonance imaging (MRI) and showed that the extensor mechanism consisted of a single tendon from the quadriceps muscle belly traveling over the trochlea and inserting onto the tibial tubercle. On MRI of the left knee, the extensor mechanism had slipped out of the trochlea and was resting on the lateral side of the femur (Figure 2). The remaining ligamentous structures were intact.

In this patient, lateral dislocation of the abnormal extensor mechanism was diagnosed on the left lower extremity. The patient was brought to the operating room, and the extensor mechanism, which was lateral to the lateral femoral condyle, was exposed from the level of the quadriceps muscle belly down to the tibial tubercle. The vastus lateralis was released off the iliotibial band at the intermuscular septum, and the lateral release was continued to the level of the tibial tubercle. After the release, the extensor mechanism was reducible and resting in the trochlea. However, when the knee was flexed to 90°, the tendon would dislocate laterally. An area of the medial capsular thickening was ellipsed and plicated and imbricated onto the medial side of the tendon with a pants-over-vest repair. A Roux-Goldthwait procedure was then performed where the distal portion of the tendon was split in half longitudinally. The lateral portion was detached from the tibial tubercle, mobilized underneath the medial portion, and sutured onto the periosteum medial to the tibial tubercle. After the procedure, the knee was placed through range of motion, and the extensor mechanism was found to be stable without evidence of lateral dislocation. Postoperatively, the patient was placed in a long leg cast at 15° of flexion and was made non–weight-bearing for 6 weeks.

After 6 weeks, the patient was transitioned to a brace and started physical therapy for quadriceps strengthening. The patient was made weight-bearing as tolerated. Three months after his surgery, the patient was ambulating painlessly with a stable gait. He had no appreciable limp. The patient was able to achieve full active flexion of the knee with approximately a 10° extensor lag. At his 6-month follow-up, his range of motion and strength continued to improve, and he had a 5° extensor lag with normal heel-toe gait. Finally, at his 10-month follow-up, the patient continued to have no knee pain with no instability events. Physical examination demonstrated strong knee extension grossly equal to the contralateral side. He still had a 5° extensor lag, but could run, jump, and play without any issues.

## Discussion

Congenital absent or small patella is a rare condition that may be associated with a spectrum of other malformations, each with a unique presentation of skeletal or extraskeletal phenotypes.^7,8,9,10,11^ Isolated absent patella has been reported without the presence of other abnormalities in only a few occurrences. The earliest study to report this condition was by Kutz^12^ in 1949 who described a 9-year-old girl in whom the absent patella was only discovered after a routine physical examination. The patient had no fingernail abnormalities with a normal medical history. Duygun et al^3^ discussed a male patient with bilateral absent patella. Full blood count was normal, and no renal impairments were noted. Jerome et al^4^ examined a patient with congenital bilateral absent patella with prominent femoral condyles and a bilateral fixed-flexion deformity. Fingernail, renal, blood count, and abdominal ultrasonography findings were absent. Finally, Varghese noted a bilateral congenital patellar aplasia with agenesis of the distal third of the quadriceps extensor mechanism.^1^

Treatment of congenital absent patella is dependent on the degree of aplasia, involvement of the extensor mechanism, and effect on leg functionality. Nonsurgical interventions include physiotherapy, splinting, or bracing treatment for the affected knee.^13^ Varghese et al performed a femur osteotomy to compensate for the flexion deformity and to reduce the distance of the rectus femoris to the knee, restoring quadriceps function.^2^ By contrast, Jerome et al^4^ performed a hamstring lengthening instead to correct the flexion deformity. Total knee arthroplasty may also be considered depending on the patient's quality of life and level of pain.^14,15^ In our case, the patient presented with a lateral dislocation of his left extensor mechanism without a flexion contracture. To address the repeated dislocation, we performed a lateral release. When the extensor mechanism was still found to be dislocating laterally during passive range of motion, a medial plication and Roux-Goldthwait procedure was added on, and the extensor mechanism was found to be reduced and stable afterward. The patient was walking 6 weeks after surgery and was participating in sports 10 months later without any issues.

## Conclusion

To our knowledge, this is the first reported case of bilateral absent patellas without associated congenital anomalies with a lateral dislocation of the extensor mechanism in the absence of a flexion contracture. Nonsurgical management with physical therapy failed for the patient, and he underwent surgery on the extensor mechanism, which included a lateral release, followed by a medial plication and Roux-Goldthwait procedure. The patient did well postoperatively with improved extensor mechanism strength, improved knee range of motion, and a return to sports at 10 months.
